# Christmas "Outward and Visible"

**Published:** 1889-12-28

**Authors:** 


### The Doctor's Pulpit.

### CHRISTMAS, "OUTWARD AND VISIBLE."

If pleasures are to be such as all men can share in, it is almost imperative that they speak to the eye or the ear, or at any rate to the taste. If they are to be quite universal, they must appeal to all three senses, and perhaps to more. Some men are religious; all are not. Some love music or beautiful sights; all do not. But every man, woman, and child born into the world both can and must eat. Eating is, therefore, perhaps almost the one exclusive thing in which all men alike can unite, and unite with pleasure. The instinct that has made men, women, and families meet and eat together at Christmastide is not the unworthy and unintellectual feeling that many consider it. On the contrary, there is in it the ring of true universality. That the meeting and the eating be of a joyous character is necessary to the full external manifestation of the true spirit of Christmas.

There is no ridding ourselves of our physical natures, at least, in this world. If a man wants to look at his neighbour he must have eyes to look with, or he cannot see him; and his neighbour must have a material body or he cannot be seen. If music is to delight and ravish the soul, it must enter by the bodily ear, or in our present condition it will not be heard. If the body is to be sustained in strength, the intellect in clearness, and the soul in majesty, it is absolute necessary that the mouth should eat and the stomach digest. To despise or ignore the body, and to think lightly of its wholesome pleasures is unscientific and absurd, and it is certainly unchristian.

We English are great believers in the "outward and visible," what we call "the real" and "the practical." It is not a bad quality. Thoughts and speculations which can be brought in from the wide-ranging fields of the ideal, and yoked to the cars of practical experimentation, lose nothing in actuality, and gain much in credit. When Christmas shows itself in bright adornments of home and church, in happy gifts of friend and lover, in cordial greetings and welcoming smiles, and in tables burdened with festive cheer and surrounded by faces that beam with joy, there is no need for rhetoric or logic to convince the world that a season of happiness is in full and swelling tide. The delights of Christmas are innocent, and they are rational and real. With us, moreover, they are peculiarly English. They are old, too, linking the present with the past, and keeping up the continuity, not only of religious feeling, but of national sentiment. It is probable that to-day the innermost hearts of all who sit round the Christmas table differ scarcely at all from those that surrounded the Christmas board in the days of Elizabeth, or even in the much remoter times of Richard Cœur de Lion.

Our people have always been fashioned out of the stuff that can be easily seen. Their character has been wont to express itself in deeds rather than in words; in lives rather than in school logic. The Christmas holly may seem to many to look sombre instead of bright. But it is not the dark green hues of the velvet leaves that are in request so much as the brilliant scarlet of the berries—a scarlet made all the more conspicuous and warm by its emerald setting. The holly is a solid and enduring kind of decoration. Not perhaps so graceful as some of the trailing plants of more tropical climes, but eminently in keeping with the national character. It is not without its stern prickles, that draw blood from unwary and too impetuous fingers. Therein it is a very fitting type of the tough and stern but not unhandsome Briton, who, placid as he ordinarily looks, can, without difficulty, make himself felt in ways that are not always pleasant. A room decorated with a profusion of abundantly-berried holly creates Christmas at a single stroke.

It is a pity that the mistletoe is losing favour with so many; and it is specially a pity that the merry and entirely wholesome custom with which it was associated is rapidly becoming a thing of the past. People bred in the country can remember with a half-bashful pleasure the gleeful amusement associated with the little bough; an amusement which was all the more delightful because in special instances much tenderer sentiments were allowed an exquisite moment's indulgence. But youthful cousins and family friends are all too dignified nowadays for the innocent simplicities of the country life of twenty years ago. It is certain they are not any wholesomer in disposition and habit, and it seems still more certain that they are not half so happy. It is a terrible thing to be so prudish that one cannot be simple and natural. London, and wealth, and pompous vanity have much to answer for.

The dinner is the central and supreme moment of all the Christmas delights, at any rate in England. "What gluttonous animals!" say our foreign critics. Even our fellow-citizens over the Scotch border deride the festivities of the Christmas Day and console themselves with unlimited haggis, Scotch bun, and whisky on the first day of the New Year instead. But we are gloriously defiant of all criticism of our Christmas roast beef and pudding, whether it comes from the land of frogs or from the mountainous regions of the whisky-stills. Deep within our innermost hearts there is a homely conviction that Christianity means much more than a few theological dogmas, or the attendance upon public worship on Sundays. All our fellow-countrymen have a kind of feeling that, however ignorant and careless they may sometimes be about the performance of religious duties, they have a certain indefeasible connection with and a personal share in the simplest, wholesomest, and noblest religion the world has ever seen. Christmas, they believe, celebrates a fact, a fact which, if the accepted interpretation of it be the true one, cannot be without large and beneficent significance for every child of man. Accordingly, at Christmas every man and woman, for the time at any rate, puts away his doubts, forgets his indifference, and even his wilful sin, and joins in the general happiness which dawned upon the world with the birth of the God-man. It is a right worthy and a right rational thing to do. If any sour fanatic thinks this view of the divine comprehensiveness dangerously and wickedly lax, let him remember what was once said about "Many who are to come from the east and from the west, from the north and from the south, to sit down in the new kingdom"; whilst the ungenerous and ill-conditioned "children of the kingdom" are to be "cast out." We only say this as showing that we are not without high warrant in claiming for all who spontaneously and heartily enter into the outward, visible, and common pleasures of the Christmas season a portion of that divine grace which would fain include everyone of them within its ample liberality.

The "outward and the visible" are never to be despised. When they express, as they certainly do in England, the assured conviction that the season is joyous, and rationally so; that the event which has been celebrated nearly nineteen hundred times is as worthy of celebration to-day as it was on its first anniversary, then they are to be welcomed with all possible warmth, and entered into with the utmost spontaneity of healthy and uncorrupted nature. The day is for ever blessed on which Jesus Christ was born. It is every man's annual holiday; and it is well that it should be celebrated in such ways that every man shall find himself entirely at home in the midst of its simple and common but universal delights.

